# Transcription factor 7-like 2 gene- smoking interaction on the risk of diabetic nephropathy in Chinese Han population

**DOI:** 10.1186/s41021-021-00194-2

**Published:** 2021-06-30

**Authors:** Peng Xue, Haihong Cao, Zhimin Ma, Ying Zhou, Nian Wang

**Affiliations:** Department of endocrinology, the Affiliated Suzhou Science and Technology Town Hospital of Nanjing Medical University, No.1 Lijiang Road, Suzhou New District, Suzhou, Jiangsu Province China

**Keywords:** Interaction, Transcription factor 7-like 2, Polymorphisms, Diabetic nephropathy, Transcription factor 7-like 2

## Abstract

**Objectives:**

To evaluate the relationship between transcription factor 7-like 2 (*TCF7L2*) gene polymorphism and diabetic nephropathy (DN) risk, as well as the effect of gene-environment interactions on DN risk in Chinese Han population.

**Methods:**

The Hardy-Weinberg equilibrium (HWE) and the relationship between *TCF7L2* gene single nucleotide polymorphism (SNPs) and DN susceptibility were evaluated by SNPStats. The interaction among four SNPs and environmental factors were tested by generalized multifactor dimensionality reduction (GMDR). The consistency of cross validation, accuracy of test balance and sign test were calculated to evaluate the interaction of each selection. The logistic regression was used to test the interaction between rs7903146 and current smoking by stratified analysis.

**Results:**

Logistic regression analysis indicated that the DN risk of rs7903146-T allele carriers were obviously higher than that in CC genotype carriers (CT + TT *versus* CC), adjusted OR (95 %CI) = 1.64 (1.24–2.06). However, we also discovered that people with rs12255372, rs11196205 and rs290487 minor allele had non-significant difference risk of DN compared with people with major allele. The GMDR model found a significant two-locus model (*p* = 0.0100) including rs7903146 and current smoking, suggesting a potential gene–environment interaction between rs7903146 and current smoking. Compared with never smokers with rs7903146- CC genotype, current smokers with rs7903146- CT or TT genotype had the highest DN risk. After covariate adjustment, OR (95 %CI) was 2.15 (1.58–2.78).

**Conclusions:**

We found a significant relationship of rs7903146-T alleles, and the interaction between rs7903146-T and current smoking with increased DN risk.

## Introduction

Diabetic nephropathy (DN) is one of the most common chronic and progressive complications of diabetes. DN is associated with higher cardiovascular incidence rate and mortality and is the most common cause of end-stage renal disease (ESRD) in the world [1, 2]. DN is also one of the important risk factors for the occurrence and development of chronic kidney disease and chronic cardiovascular disease [3], and one of the main causes of death in the world [4]. The pathogenesis of DN is complex and multifactorial, and the specific mechanism is still unclear. However, previous studies [5, 6] have shown that the risk factors of DN include genetic factors and environmental factors, such as hypertension, hyperglycemia and hyperlipidemia.

The transcription factor 7-like 2 (*TCF7L2*), is a highly variable transcription factor, which plays an important role in regulating insulin secretion and maintaining glucose homeostasis in pancreatic β cells [7]. In addition, *TCF7L2* protein, which is encoded by the *TCF7L2* gene, is involved in regulation of endothelial cell growth and smooth muscle cell proliferation, and then affects vascular remodeling [8]. *TCF7L2* gene is located on chromosome 10q25.3, which contains 215,863 bases, including 17 exons, encoding 596 amino acids. Previous epidemiological studies have indicated that the *TCF7L2* gene single nucleotide polymorphisms (SNPs) were associated with common diseases, including type 2 diabetes mellitus (T2DM) [9] and DN [10–12]. The study on the relationship between single nucleotide polymorphism of *TCF7L2* gene and DN risk in Chinese Han populations is very limited. Additionally, DN development has been proved to be the outcome of complicated interaction among genetic and environmental factors, up to now, limited researches focused on the association of interaction between *TCF7L2* gene SNPs and environmental factors with DN risk. Therefore, the purpose of this study is to evaluate the relationship between *TCF7L2* gene polymorphism and DN risk, as well as the effect of gene-environment interactions on DN risk in Chinese Han population.

## Participant selection and methods

### Study population

Subjects were recruited continuously from our Hospital between June 2013 and July 2019. A total of 1083 subjects with an average age of 67.5 ± 13.9 years were selected, including 358 T2DM with DN cases and 720 T2DM without DN controls. All participants were selected from T2DM patients, and those T2DM with DN patients were included in case group, and those T2DM without DN patients were included in the control group. The diagnosis of T2DM and DN was diagnosed by two pathologists in our Hospital according to the World Health Organization diagnostic criteria [13]. Those patients with poor glycemic control, overt nephropathy, significant heart failure, treated by chemotherapy or radiotherapy (to ensure the accuracy of our information collection) or had any kinds of cancers were removed. The control group was matched to patients by sex, age and ethnic background, and those participants with family history of DN or others kidney disease were excluded. Current cigarette smoking was defined as those who self-reported smoking cigarettes at least once a day for 1 year or more.

### Genotyping methods

The *TCF7L2* gene polymorphism (rs7903146, rs12255372, rs290487 and rs11196205) genotyping was performed by polymerase chain reaction (PCR) and following restriction fragment length polymorphism (RFLP). According to instructions of DNA Blood Mini Kit (Qiagen, Hilden, Germany), 3 ml EDTA-processed blood samples were extracted from all participants for DNA extraction, and DNA was preserved at -20 °C before use. All primers applied in our research are shown in Table 1.

**Table 1:** Description and primer sequences used for genotyping for 4 SNPs within TCF7L2 gene

SNP ID	Chromosome	Functional Consequence	Nucleotide substitution	Primer sequences
rs7903146	10:112,998,590	Intron variant	C > T	F: 5′- CAAATTCATGGGCTTTCT − 3′ R: 5′- CCTTCCCTGTAACTGTGG − 3′
rs11196205	10:113,047,288	Intron variant	G > T	F: 5′- GAAAGT TCTCAACATTTATAACTTCG-3′ R: 5′- TTTGCCCAATAATATGCCATGAAA-3′
rs290487	10:113,149,972	Intron variant	C > T	F: 5′- GCTGCCATATTGTTTACT − 3′ R: 5′- ATGATTTGTACTGGGTTG − 3′
rs12255372	10:113,049,143	Intron variant	G > T	F: 5′- CTTGAGGTGTACTGGAAACTAAGGC-3′ R: 5′- CTGTCTATTTGGCATTCAAATGGA-3′

### Statistical analysis

In our study, the mean and standard deviations (SDs) were calculated for continuous variables with normal distribution, and the percentages were calculated for categorical variables. The χ2 test was used for comparison for percentages and *t* test was used for comparison of means and SDs. The Hardy-Weinberg equilibrium (HWE) and the relationship between *TCF7L2* gene SNPs and DN susceptibility were evaluated by SNPStats (https://www.snpstats.net/). The interaction among four SNPs and environmental factors were tested by generalized multifactor dimensionality reduction (GMDR) [14]. The consistency of cross validation, accuracy of test balance and sign test were calculated to evaluate the interaction of each selection. The logistic regression was used to test the interaction between rs7903146 and current smoking by stratified analysis.

## Results

A total of 1083 subjects with an average age of 67.5 ± 13.9 years were selected, including 358 T2DM with DN cases and 720 T2DM without DN controls. The characteristics of subjects stratified by case and control are shown in Table 2. No statistically significant differences were observed between cases and controls in terms of age and proportion of males. The body mass index (BMI), smoking and alcohol drinking, fasting plasma glucose (FPG), duration of diabetes and hypertension rate in cases were significantly higher than that in controls.

**Table 2:** General characteristics of 1083 study participants in case and control group

Variables	T2DM with DN patients (*n* = 358)	T2DM without DN controls *(n* = 720)	*p-*values
Age (year), means ± SD	68.3 ± 14.2	67.3 ± 13.6	0.263
Males, N (%)	213 (59.5)	419 (58.2)	0.682
BMI (kg/m^2^), means ± SD	25.4 ± 7.4	23.5 ± 6.9	0.000035
Smoking, N (%)	119 (33.2)	188 (26.1)	0.015
Alcohol drinking, N (%)	140 (39.1)	224 (31.1)	0.0089
FPG (mmol/l)	9.3 ± 2.9	8.6 ± 3.1	0.000378
Duration of diabetes	10.8 ± 6.4	9.6 ± 6.7	0.005034
Hypertension, N (%)	103 (28.8)	162 (22.5)	0.0243

BMI: body mass index; T2DM: type 2 diabetes mellitus; FPG: fasting plasma glucose

HWE test was performed for controls, and we found that all genotypes were distributed according to HWE (all *p* values more than 0.05). The allele frequency of rs7903146-T and in DN group was significantly higher than that in control group (28.9 % vs19.7 %). Logistic regression analysis indicated that the DN risk of rs7903146-T allele carriers was obviously higher than that in CC genotype carriers (CT + TT *versus* CC), adjusted OR (95 %CI) = 1.64 (1.24–2.06). However, we also discovered that people with rs12255372, rs11196205 and rs290487 minor allele had non-significant difference risk of DN compared with people with major allele (Table 3).

**Table 3:** Genotype distribution and allele frequencies of four SNPs in case and control group

SNPs	Genotypes and alleles	Frequencies N (%)		OR (95 %CI) *	P-values
		Control (*n* = 720)	Case (*n* = 358)		
rs12255372					
	GG	419 (58.2)	191 (53.4)	Ref	
	GT	260 (36.1)	140 (39.1)	1.23 (0.90–1.66)	
	TT	41 (5.7)	27 (7.5)	1.38 (0.81–1.97)	
	G	1098 (76.02	522 (72.9)	Ref	
	T	342 (23.8)	194 (27.1)	1.26 (0.88–1.69)	
HWE test for controls					0.936
rs7903146					
	CC	469 (65.1)	185 (51.7)	Ref	
	CT	218 (30.3)	139 (38.8)	1.59 (1.21–1.99)	
	TT	33 (4.6)	34 (9.5)	1.82 (1.27–2.43)	
	C	1156 (80.3)	509 (71.1)	Ref	
	T	284 (19.7)	207 (28.9)	1.64 (1.24–2.06)	
HWE test for controls					0.240
rs11196205					
	GG	427 (59.3)	191 (53.4)	Ref	
	GT	256 (35.6)	139 (38.8)	1.28 (0.83–1.76)	
	TT	37 (5.1)	28 (7.8)	1.42 (0.78–2.09)	
	G	1110 (77.1)	521 (72.8)	Ref	
	T	330 (22.9)	195 (27.2)	1.30 (0.81–1.87)	
HWE test for controls					0.864
rs290487					
	TT	473 (65.7)	215 (60.1)	Ref	
	TC	219 (30.4)	123 (34.4)	1.30 (0.96–1.67)	
	CC	28 (3.9)	20 (5.6)	1.46 (0.90–2.05)	
	T	1165 (80.9)	553 (77.2)	Ref	
	C	275 (19.1)	163 (22.8)	1.33 (0.94–1.72)	
HWE test for controls					0.674

*Adjusted for gender, age, BMI, smoking, alcohol drinking, FPG, duration of diabetes and hypertension

The GMDR model was used to evaluate the effect of SNP-SNP and gene- environmental factors interaction between 4 SNPs on DN risk. Table 4 shows the GMDR analysis results of SNP-SNP interaction and gene-current smoking interaction. We found a significant two-locus model (*p* = 0.0100) including rs7903146 and current smoking, suggesting a potential gene–environment interaction between rs7903146 and current smoking. The cross-validation consistency of two-locus model was 9 of 10, and the testing accuracy was 0.5985. Compared with never smokers with rs7903146- CC genotype, current smokers with rs7903146- CT or TT genotype had the highest DN risk. After covariate adjustment, OR (95 %CI) was 2.15 (1.58–2.78) (Table 5).

**Table 4:** GMDR analysis on the best SNP–SNP and gene- current smoking interaction models

Locus no.	Best combination	Cross-validation consistency	Testing balanced accuracy	p-values^*^
SNP- SNP interactions *				
2	(1, 3)	8/10	0.5451	0.3770
3	(1, 3, 2)	6/10	0.5577	0.3240
4	(1, 3, 2, 4)	7/10	0.4960	0.6241
Gene- current smoking interactions **				
2	(1, 5)	9/10	0.5985	0.0107
3	(1, 5, 3)	7/10	0.5622	0.3770
4	(1, 5, 3, 2)	6/10	0.5577	0.1719
5	(1, 5, 3, 2, 4)	7/10	0.5212	0.4258

*Adjusted for gender, age, BMI, smoking, alcohol drinking, FPG, duration of diabetes and hypertension

**Adjusted for gender, age, BMI, alcohol drinking, FPG, duration of diabetes and hypertension

rs7903146, rs12255372, rs290487, rs11196205 and current smoking were symbolized as 1–5, respectively

**Table 5:** Stratified analysis for rs7903146 and current smoking on DN risk using logistic regression

rs7903146	Current smoking	OR (95 % CI) ^*^	*P*-values
CC	No	1.00	-
CT or TT	No	1.37 (1.03–1.55)	0.030
CC	Yes	1.58 (1.16–2.02)	0.002
CT or TT	Yes	2.15 (1.58–2.78)	< 0.001

*Adjusted for gender, age, BMI, alcohol drinking, FPG, duration of diabetes and hypertension

## Discussion

In this study, we evaluated the effect SNPs of *TCF7L2* on DN risk, and we found that the DN risk was obviously higher in **rs7903146-T** allele carriers than that in CC genotype carriers, However, we also discovered that people with rs12255372, rs11196205 and rs290487 minor allele had non-significant difference risk of DN compared with people with major allele. Previous studies have reported a statistically significant association between *TCF7L2* gene polymorphism and DN and coronary atherosclerosis [15, 16]. In recent years, the association between *TCF7L2* gene polymorphism and DN has been reported in several studies [17–20]. However, there are few studies on the association between *TCF7L2* mutation and DN risk in Chinese population. *TCF7L2* is a transcription factor containing DNA binding domain. Its coding gene is widely expressed in many organs of human body, mainly in adipose tissue and human pancreatic beta cells [21]. Previously, two studies conducted by Buraczynska et al. [17, 22] has verified that the rs7903146-T allele of *TCF7L2* gene was significantly associated with DN, especially in the early stage of diabetes. Zhuang et al. [18] carried out a case-control study in Chinese population, and found that *TCF7L2* rs7903146 polymorphism had a significant impact on the susceptibility to DN in Chinese Han population, but rs290487 had no statistical association with DN. This was consistent with conclusions from Lewis et al. [23], Hussain et al. [12] and Sale et al. [24], But Fu et al. [25] concluded inconsistent results on relationship between *TCF7L2* rs7903146 polymorphism and DN risk, in this study, just 248 DN patients were included in the analysis, so the limited sample size maybe the reason for inconsistent results between this study and our study. A case-control study conducted by Bodhini et al. [26] showed that rs12255372 polymorphism in *TCF7L2* gene was associated with type 2 diabetes and DN, but its association with DN was affected by diabetes. However, study suggested that minor allele of rs12255372 had non-significant impact on risk of DN.

DN susceptibility was influenced by too many risk factors, involving genetic factors, environmental factors, gene- gene and gene- environment interactions. In current study, just one gene was investigated, therefore we could not estimate the gene-gene interaction on DN risk, just the SNP-SNP interaction was investigated. As we all known that smoking was a risk factor for DN risk [27, 28], previous study has reported a significant gene- environment interaction between *MTHFR C677T* polymorphism (rs1801133) and smoking on susceptibility to DN in Chinese men with T2DM. But to date, no study focused in the impact of interaction between *TCF7L2* gene and smoking on DN risk. In this study, the current smoking rate was higher than that of control group, which means that the current smoking was a risk factor for DN susceptibility. Therefore, we also conducted *TCF7L2* gene-environment interaction between fours SNPs and current smoking using GMDR model. We found a significant two-locus model including rs7903146 and current smoking. Compared with never smokers with rs7903146- CC genotype, current smokers with rs7903146- CT or TT genotype had the highest DN risk. The exact interaction mechanism for this gene- environment interaction is still unclear, but we believe that TCF7L2 gene and current smoking are related to DN risk factors, which might be the basis of gene- environment interaction.

This research has some limitations. Firstly, just four SNPs of the *TCF7L2* gene were selected for genotyping, and more SNPs within *TCF7L2* gene should be included in future studies. Secondly, more environmental factors should be included in the GMDR model to investigate the gene-environment interaction. Lastly, the participants included in this study were all Chinese Han population, and the results obtained in our study should be verified in different ethnicities in China or different countries.

## Conclusions

In summary, our research shows that rs7903146- T allele, interaction between rs7903146 and current smoking are all associated with increased susceptibility to DN. Although, previous studies have reported the relationship between rs7903146- T allele and DN risk, but these studies did not performed gene- smoking interaction analysis, and which was the new or additional finding on DN genetic association investigation.

## Data Availability

Not applicable.

## Abbreviations

TCF7L2: Transcription factor 7-like 2
SNP: Single nucleotide polymorphism
DN: Diabetic nephropathy
ESRD: end-stage renal disease
T2DM: type 2 diabetes mellitus
PCR: polymerase chain reaction
RFLP: restriction fragment length polymorphism
HWE: Hardy-Weinberg equilibrium
SDs: standard deviations
GMDR: generalized multifactor dimensionality reduction
FPG: fasting plasma glucose
BMI: body mass index

## References

[1] Ding Y, Choi ME. Autophagy in diabetic nephropathy. J Endocrinol. 2015;224:R15–30. 10.1530/JOE-14-0437 PMC4238413 25349246

[2] Gross JL, de Azevedo MJ, Silveiro SP, Canani LH, Caramori ML, Zelmanovitz T (2005). Diabetic nephropathy: Diagnosis, prevention, and treatment. Diabetes Care.

[3] Gnudi L, Coward RJM, Long DA (2016). Diabetic nephropathy: perspective on novel molecular mechanisms. Trends Endocrinol Metab.

[4] Kos I, Prkacin I (2014). Diabetic nephropathy as a cause of chronic kidney disease. Acta Med Croatica.

[5] Tziomalos K, Athyros VG (2015). Diabetic nephropathy: new risk factors and improvements in diagnosis. Rev Diabet Stud.

[6] Papanas N, Ziegler D (2015). Risk factors and comorbidities in diabetic neuropathy: an update 2015. Rev Diabet Stud.

[7] Zhou Y, Park SY, Su J, Bailey K, Ottosson-Laakso E, Shcherbina L, Oskolkov N, Zhang E, Thevenin T, Fadista J, Bennet H, Vikman P, Wierup N, Fex M, Rung J, Wollheim C, Nobrega M, Renström E, Groop L, Hansson O (2014). TCF7L2 is a master regulator of insulin production and processing. Hum Mol Genet.

[8] Ngwa EN, Sobngwi E, Atogho-Tiedeu B, Noubiap JJ, Donfack OS, Guewo-Fokeng M, Mofo EP, Fosso PP, Djahmeni E, Djokam-Dadjeu R, Evehe MS, Aminkeng F, Mbacham WF, Mbanya JC (2015). Association between the rs12255372 variant of the TCF7L2 gene and obesity in a Cameroonian population. BMC Res Notes.

[9] Zhou KC, Liu HW, Wang C, Fu YJ, Jin F (2019). Association of transcription factor 7-like 2 (TCF7L2) gene polymorphism with type 2 diabetes mellitus in Chinese Korean ethnicity population. Med (Baltim).

[10] Morgan MF, Salam RF, Rady NH, Alnaggar ARLR, Ammar SH, Ghanem NS (2020). The Association of Transcription Factor 7 like 2 Gene Polymorphism with Diabetic Nephropathy in patients with type 2 diabetes mellitus. Curr Diabetes Rev.

[11] Araoka T, Abe H, Tominaga T, Mima A, Matsubara T, Murakami T, Kishi S, Nagai K, Doi T (2010). Transcription factor 7-like 2 (TCF7L2) regulates activin receptor-like kinase 1 (ALK1)/Smad1 pathway for development of diabetic nephropathy. Mol Cells.

[12] Hussain H, Ramachandran V, Ravi S, Sajan T, Ehambaram K, Gurramkonda VB, Ramanathan G, Bhaskar LV (2014). TCF7L2 rs7903146 polymorphism and diabetic nephropathy association is not independent of type 2 diabetes–a study in a south Indian population and meta-analysis. Endokrynol Pol.

[13] World Health Organization (1999). Definition, diagnosis and classification of diabetes mellitus and its complications. Report of a WHO Consult. Part 1: Diagnosis and Classification of Diabetes Mellitus.

[14] Lou XY, Chen GB, Yan L, Ma JZ, Zhu J, Elston RC, Li MD (2007). A generalized combinatorial approach for detecting gene-by-gene and gene-by-environment interactions with application to nicotine dependence. Am J Hum Genet.

[15] Savic D, Ye H, Aneas I, Park SY, Bell GI, Nobrega MA (2011). Alterations in TCF7L2 expression define its role as a key regulator of glucose metabolism. Genome Res.

[16] Muendlein A, Saely CH, Geller-Rhomberg S, Sonderegger G, Rein P, Winder T, Beer S, Vonbank A, Drexel H (2011). Single nucleotide polymorphisms of TCF7L2 are linked to diabetic coronary atherosclerosis. PLoS ONE.

[17] Buraczynska M, Swatowski A, Markowska-Gosik D, Kuczmaszewska A, Ksiazek A (2011). Transcription factor 7-like 2 (TCF7L2) gene polymorphism and complication/ comorbidity profile in type 2 diabetes patients. Diabet Res Clin Pract.

[18] Zhuang Y, Niu F, Liu D, Sun J, Zhang X, Zhang J, Guo S (2018). Associations of TCF7L2 gene polymorphisms with the risk of diabetic nephropathy: A case-control study. Med (Baltim).

[19] Fan Z, Cai Q, Chen Y, Meng X, Cao F, Zheng S, Guo J (2016). Association of the Transcription Factor 7 Like 2 (TCF7L2) Polymorphism With Diabetic Nephropathy Risk: A Meta-Analysis. Med (Baltim).

[20] Wu LS, Hsieh CH, Pei D, Hung YJ, Kuo SW, Lin E (2009). Association and interaction analyses of genetic variants in ADIPOQ, ENPP1, GHSR, PPARgamma and TCF7L2 genes for diabetic nephropathy in a Taiwanese population with type 2 diabetes. Nephrol Dial Transplant.

[21] Osmark P, Hansson O, Jonsson A, Rönn T, Groop L, Renström E (2009). Unique splicing pattern of the TCF7L2 gene in human pancreatic islets. Diabetologia.

[22] Buraczynska M, Zukowski P, Ksiazek P, Kuczmaszewska A, Janicka J, Zaluska W (2014). Transcription factor 7-like 2 (TCF7L2) gene polymorphism and clinical phenotype in end-stage renal disease patients. Mol Biol Rep.

[23] Lewis JP, Palmer ND, Hicks PJ, Sale MM, Langefeld CD, Freedman BI, Divers J, Bowden DW (2008). Association analysis in African Americans of European derived Type 2 diabetes single nucleotide polymorphisms from whole genome association studies. Diabetes.

[24] Sale MM, Smith SG, Mychaleckyj JC, Keene KL, Langefeld CD, Leak TS, Hicks PJ, Bowden DW, Rich SS, Freedman BI (2007). Variants of the transcription factor 7-like 2 (TCF7L2) gene are associated with type 2 diabetes in an African- American population enriched for nephropathy. Diabetes.

[25] Fu LL, Lin Y, Yang ZL, Yin YB (2012). Association analysis of genetic polymorphisms of TCF7L2, CDKAL1, SLC30A8, HHEX genes and microvascular complications of type 2 diabetes mellitus. Zhonghua Yi Xue Yi Chuan Xue Za Zhi.

[26] Bodhini D, Chidambaram M, Liju S, Prakash VG, Gayathri V, Shanthirani CS, Ranjith U, Anjana RM, Mohan V, Radha V (2015). Association of TCF7L2 Polymorphism with Diabetic Nephropathy in the South Indian Population. Ann Hum Genet.

[27] Han Q, Wang S, Zhang J, Zhang R, Guo R, Wang Y, Li H, Xu H, Liu F (2018). The association between cigarette smoking and diabetic nephropathy in Chinese male patients. Acta Diabetol.

[28] Liao D, Ma L, Liu J, Fu P. Cigarette smoking as a risk factor for diabetic nephropathy: A systematic review and meta-analysis of prospective cohort studies. PLoS One. 2019;14(2):e0210213. 10.1371/journal.pone.0210213 PMC6361430 30716100

